# The Role of Chinese Herbal Therapy in Methamphetamine Abuse and its Induced Psychiatric Symptoms

**DOI:** 10.3389/fphar.2021.679905

**Published:** 2021-05-10

**Authors:** Lin Chen, Qin Ru, Qi Xiong, Mei Zhou, Kai Yue, Yuxiang Wu

**Affiliations:** ^1^ Department of Health and Physical Education, Jianghan University, Wuhan, China; ^2^ Wuhan Institutes of Biomedical Sciences, Jianghan University, Wuhan, China

**Keywords:** addiction, psychiatric impairment, neurobiological mechanisms, chinese herbal medicines, METH abuse

## Abstract

Repeated intake of methamphetamine (METH) leads to drug addiction, the inability to control intake, and strong drug cravings. It is also likely to cause psychiatric impairments, such as cognitive impairment, depression, and anxiety. Because the specific neurobiological mechanisms involved are complex and have not been fully and systematically elucidated, there is no established pharmacotherapy for METH abuse. Studies have found that a variety of Chinese herbal medicines have significant therapeutic effects on neuropsychiatric symptoms and have the advantage of multitarget comprehensive treatment. We conducted a systematic review, from neurobiological mechanisms to candidate Chinese herbal medicines, hoping to provide new perspectives and ideas for the prevention and treatment of METH abuse.

## Introduction

Psychostimulants, including methamphetamine (METH) and other amphetamines (AMPHs), are inferior to marijuana and have become the most diffusely used class of drugs globally (United Nations, 2020). The abuse of METH and other AMPHs has become a serious public health problem and a growing global concern. Regardless of the person, family, country, or society, the abuse of METH has led to an obvious increase in various burdens, including the consumption of public health resources (Siefried et al., 2020).

METH can cross the blood-brain barrier and act on the central nervous system. It mainly alters neurotransmission by interfering with dopamine (DA), DA transporters (DAT), and increasing the DA concentration in the brain (Brensilver et al., 2013). Repeated use of METH leads to chronic recurrent drug dependence that is characterized by compulsive, uncontrolled drug use and intense cravings (Brensilver et al., 2013; Mizoguchi and Yamada, 2019). METH is more likely to cause psychiatric impairments than traditional opioids (Glasner-Edwards and Mooney, 2014; Eslami-Shahrbabaki et al., 2015). Related epidemiological and clinical studies have suggested that people abusing METH have a significantly increased risk of schizophrenia (Callaghan et al., 2012) and are more prone to cognitive impairment (Wagner et al., 2013; Potvin et al., 2018; Mizoguchi and Yamada, 2019), depression (Marshall and Werb, 2010), anxiety (McKetin et al., 2016), and suicide attempts (Glasner-Edwards et al., 2008).

### The Neurobiological Mechanisms Involved in Methamphetamine Abuse

METH indirectly activates DA, 5-hydroxytryptamine (5-HT), glutamate (Glu), and adrenaline receptors by increasing monoamine transmitters in the synaptic cleft, producing a series of physiological effects (Cruickshank and Dyer, 2009). In the central nervous system, METH can indirectly stimulate adrenaline receptors, causing increased alertness, vitality, and attention. METH also induces the release of DA, which can induce pleasure. Working memory and reasoning ability can be enhanced by activating D1 receptors and α_2_ adrenergic receptors in the prefrontal cortex (PFC); excited 5-HT receptors have antianxiety effects, making people feel relaxed and confident (Berridge, 2006; Weinshenker and Schroeder, 2007). These positive drug-induced experiences are the main reasons why METH is widely abused. However, long-term heavy use of METH can produce significant toxic effects on the nervous system, which not only leads to abnormal brain function but also damages the brain structure. At the beginning of this manuscript, we reviewed the neurobiological mechanisms of METH addiction, including cognitive dysfunction, anxiety, depression, oxidative stress, and inflammation. The specific neurobiological mechanisms of METH addiction are summarized in Table 1.

**TABLE 1:** The effect of METH on neurotransmitters, their addictive effects, and/or psychiatric impairment.

		Addictive effects and/or major psychiatric impairment	Neurotransmitters	Receptor or target	Effect
METH impairment	Addictive effects	METH dependence	DA	D1, D2, D3, DAT	DA release and reuptake imbalances; DA receptor activation
			5-HT	SERT	Release of 5-HT; increases synthesis and release of DA
			Glu	D1	Increased glu and DA release via D1 receptor-mediated glutamate disinhibition
			GABA	GABAA	Inhibits the GABAB receptor signaling pathway
	Neuronal injury	Memory and cognitive deficits	DA	D1, D2, D3, DAT, HCN1	DA release and reuptake imbalances and apoptosis pathways activation
			Glu	mGluR5 and GluNR2B	Deduced glutamate homeostasis, decreased expression of mGluR5 and GluNR2B
		Anxiety and depression	Monoamine neurotransmitters	Monoamine neurotransmitters receptor	Monoamine neurotransmitters depletion
				Apoptotic signaling pathways	Apoptosis
				Mitochondria and endoplasmic reticulum	Stress cascading activation
			Neuronal (damage)	Microglia and astrocytes	Inflammation and overactivation
				BDNF and NGF	Neurotrophic action
				Toxic dopamine quinone, oxygen free radicals, hydrogen peroxide, and increased ROS in neuron cells	Oxidative stress

METH = methamphetamine; R = receptor; 5-HT = 5-hydroxytryptamine; D or DA = dopamine; DAT = Dopamine transporter; SERT = 5-HT transporter; GABA = gamma-aminobutyric acid; Glu = glutamate; NR2B = N-methyl-d-aspartate receptor subtype-2B; HCN1 = hyperpolarization-activated and cyclic nucleotide-gated cation 1; BDNF = brain derived neurotrophic factor; NGF = nerve growth factor.

### Methamphetamine and Addiction

Chronic METH abuse elicits compulsive craving and dependency (Miner et al., 2019). The neurobiological mechanism of METH addiction has been studied for several years. The ‘incentive‐sensitization theory’ of addiction is the most widely recognized classical theory about METH‐induced behavioral hypersensitization and rewarding (Siefried et al., 2020). METH stimulates the brain's reward system, leading to drug-related overstimulation, compulsive motivation, and excessive drug intake (Cruickshank and Dyer, 2009). The neural circuits involved in METH addiction are extensive and complex, involving many brain nuclei and brain regions, neurotransmitters, and protein mediators (Kauer and Malenka, 2007). Among them, the DA system is the most studied, and other systems are also involved to varying degrees.

The balanced state of release and reuptake of DA is an important prerequisite for DA to participate in various physiological activities. METH inhibits DA reuptake by the DAT and enhances synaptic DA release. Therefore, METH can activate DA receptors in the brain reward system to induce reward‐motivated behavior (Volz et al., 2007). Different DA receptors play different roles in METH addiction. The D1 receptor is closely involved in METH-induced drug administration, location preference, and drug-seeking behaviour, while the D2 receptor is involved in METH-mediated neurotoxicity (Carati and Schenk, 2011). Studies have also found that the D3 receptor (D3R) is closely involved in METH addiction and has also been proven to play an important role in METH‐induced hypersensitization in rats (Jiang et al., 2018). An increase in striatal D3R dopaminergic neurotransmission is associated with compulsive drug-seeking behavior in METH addicts. D3R antagonists may serve as a therapeutic tool for craving and relapse in METH addicts (Boileau et al., 2016).

In addition to DA, 5-HT is another important neurotransmitter in the processes leading to METH-induced nerve injury and addiction. METH can cause a dramatic increase and release of serotonin in the body, which is due to its indirect effect on the 5-HT transporter (SERT) (Sora et al., 2009). Moreover, the 5-HT system and the DA system interact in the mechanism of METH addiction. On the one hand, METH promotes the endogenous release of 5-HT through an independent mechanism of the 5-HT reuptake transporter (Scearce-Levie et al., 1999). On the other hand, a large amount of endogenous 5-HT can increase the synthesis and release of DA, and the regulation of the release of DA in the central ‘reward system’ may be the mechanism of the 5-HT system in METH drug addiction (Thomas et al., 2010). However, the interaction between the 5-HT and DA systems needs to be further investigated.

In addition, glutamate, as the ‘assistant’ of DA, has attracted much attention because of its involvement in the sensitization and plasticity of neurons in the central nervous system, and glutamate neurotoxicity is an important cause of pathological changes in the nervous system. During METH abuse, due to D1 receptor-mediated glutamate disinhibition in the cortical striatum, extracellular DA increases, leading to a sharp increase in glutamate in the striatum (Trudeau et al., 2014). METH can also increase the activity of glutamate neurons in the ventral tegmental area (VTA), thus inducing an increase in DA release in the nucleus accumbens (NAc) and PFC (Mark et al., 2007).

METH-induced addiction also involves gamma-aminobutyric acid (GABA) neurons and their receptor signaling pathways. METH can affect the activity of the GABAA receptor and decrease the potential induced by the GABAA receptor, which may be caused by the competitive binding of METH to the GABAA receptor (Hondebrink et al., 2013). METH can also inhibit the GABAB receptor signaling pathway of GABA neurons in the VTA region (Padgett et al., 2012). GABA receptor agonists can counteract METH-induced GABA neuron damage and conditioned positional preference (CPP) behavior in rats (Voigt et al., 2011).

### Methamphetamine and Cognitive Deficits

Cognition involves various intellectual capabilities, such as memory, attention, processing speed, and multitasking ability (Potvin et al., 2018). Cognitive abilities are necessary to function in society, and METH abuse can lead to cognitive problems that interfere with daily life (Dean et al., 2013). Previous researchers have been interested in understanding the cognitive deficits caused by METH because of its neurotoxic properties. Long-term METH abuse can cause permanent brain damage, which translates into persistent cognitive deficits. It is commonly believed that METH is an addictive drug with neurotoxic properties that damages the nervous system and induces cognitive impairment (Scott et al., 2007; Panenka et al., 2013).

To date, several cognitive deficits have been identified in METH addicts, including reaction time, working or attention memory, learning and memory, motor skills, information processing speed, and executive function deficits. Studies have shown that continuous METH use can cause medium effect-size cognitive impairment (Scott et al., 2007). A recent report also revealed that moderate impairments occur in most cognitive categories, including attention, verbal fluency, learning and memory, executive function, and visual and working memory. However, the societal consequences of METH cognitive impairment also need to be understood (Potvin et al., 2018).

METH has been found to cause abnormal changes in several neurotransmitters, such as DA overflow, leading to memory deficits (Nordahl et al., 2003). METH abuse has persistent adverse effects on the dopaminergic system, including DA release, reuptake, transport, and metabolism (Moszczynska and Callan, 2017; Anneken et al., 2018). Recent studies have shown that METH abuse can cause excessive release of DA in the PFC, activate neuronal apoptosis pathways, and eventually lead to impaired memory function (Long et al., 2017). Chronic use of antipsychotics causes downregulation of D1 receptors in the PFC, which severely damages working memory (Castner et al., 2000). Therefore, D1 receptor regulation in the PFC plays an important role in working memory and is an important target for the treatment of cognitive dysfunction (Thompson et al., 2014; Wang et al., 2019).

Prefrontal glutamatergic dysregulation may also impact recognition memory (Barker et al., 2007). Repeated METH exposure alters neuronal firing states and reduces glutamate homeostasis (Parsegian and See, 2014). METH decreases the expression of mGluR5 and GluNR2B in the cortex after two weeks of abstinence (Reichel et al., 2011). Because blocking both mGluR5-and GluNR2B-containing N-methyl-d-aspartate (NMDA) receptors impairs memory, these receptors are important in memory and cognitive function (Barker and Warburton, 2008).

### Methamphetamine and Depression

The severity of METH exposure is associated with increased rates of anxiety and depression (Glasner-Edwards et al., 2009). METH abusers with depression are more likely to alleviate depressive symptoms by taking drugs again and continue to use METH at a much higher rate than other populations. METH addicts also have significantly higher rates of depressive symptoms after withdrawal (Nakama et al., 2008). Studies have proven that the negative mood of addicts is closely associated with drug craving (Quello et al., 2005). METH abuse can lead to or worsen depressive and other psychiatric symptoms, which can increase the likelihood of further METH abuse (Glasner-Edwards et al., 2009; May et al., 2020).

Abuse of METH induces the release of neurotransmitters that cause feelings of euphoria, thereby affecting the brain's reward pathways (Glasner-Edwards and Mooney, 2014). METH induces rapid accumulation of monoamine neurotransmitters in brain synapses, which interact with DA, norepinephrine, and SERT in neurons to produce pharmacological effects (Cruickshank and Dyer, 2009). The DA level in the brain can affect emotional conditions such as depression and anxiety, leading to pathological changes such as reward effects and drug craving (Cadet and Brannock, 1998; Siefried et al., 2020). In METH addicts, long-term abuse of METH severely impairs the structure and function of the brain's monoamine transmitter system and eventually leads to the depletion of monoamine neurotransmitters in the brain (Cadet et al., 2003). METH abuse can also lead to the release of monoamine transmitters such as DA, norepinephrine, 5-HT, and other neurotransmitters (Rothman and Baumann, 2003; Panenka et al., 2013). Concretely, chronic abuse of METH can deplete DA reserves in the brain and reduce DA receptor availability (London, 2016). In summary, long-term METH abuse depletes reserves of DA in the brain and reduces the availability of DA receptors (Alex and Pehek, 2007; Ferrucci et al., 2013). METH can also have a negative impact on motor and executive function, which are usually associated with anxiety and depression (Rusyniak, 2013). Recent studies have demonstrated that METH can induce neuropathological changes through apoptotic signaling pathways in the rodent brain. Data suggest that mitochondria- and endoplasmic reticulum-mediated cascade activation is involved in METH-induced apoptosis and that neuronal apoptosis aggravates the occurrence of depression (Cadet et al., 2003).

Microglia are mainly involved in the regulation of inflammation in the central nervous system and protect the brain against injury and damage (Graeber and Streit, 2010). However, microglial overactivation can induce the release of various cytokines, reactive oxygen species, and nitrogen species, ultimately leading to neuronal damage. A study found that the inflammation induced by METH exposure may play an important role in neuronal damage (Beardsley and Hauser, 2014). Clinically, neuropsychiatric impairments, including cognitive deficits, depression, and anxiety that have been found in METH addicts, are associated with the inflammatory response (Sadek et al., 2007; Zorick et al., 2010). Elevated levels of the proinflammatory cytokines interleukin-1β (IL-1β), interleukin-2 (IL-2), interleukin-6 (IL-6), and tumor necrosis factor-α (TNF-α) in plasma were obviously associated with severe neurocognitive impairment in METH addicts (Loftis et al., 2011). It has been suggested that METH-induced neuroinflammation in the striatum may be the common basis of depression and cognitive deficits in METH addicts (Krasnova et al., 2016).

Research has shown that brain-derived neurotrophic factor (BDNF) plays an increasingly important role in anxiety and depression. BDNF is involved in the pathophysiological process of depression and plays an antidepressant role (Heyman et al., 2012; Archer et al., 2014). Physical exercise has been found to reverse the physical and neurological damage caused by METH exposure by increasing BDNF release, which is the basis for antidepressant effects (Huang et al., 2019). Studies have shown that other neurotrophic factors, such as nerve growth factor (NGF) and BDNF, also play a key role in the neurophysiological mechanisms that relieve depression (Overstreet et al., 2010; Hochstrasser et al., 2013).

### Methamphetamine and Neuronal Injury

Core mechanisms of nervous system damage caused by METH include over release of monoamine transmitters, oxidative stress, mitochondrial dysfunction, and inflammation (Krasnova and Cadet, 2009; Shin et al., 2012). These mechanisms may be the common pathological basis for METH-induced neuropsychiatric disorders such as addiction, impairments in learning, memory and cognition, anxiety, and depression, and it is necessary to highlight these mechanisms here.

High concentrations of DA in the cytoplasm produce toxic dopamine quinone, oxygen free radicals, hydrogen peroxide, and increased reactive oxygen species (ROS) in neuronal cells, resulting in oxidative stress, mitochondrial dysfunction, and damage to the presynaptic membrane (Cadet and Brannock, 1998; Lin et al., 2012). Studies have shown that tyrosine hydroxylase inhibitors inhibit DA synthesis and thus protect against neurotoxic effects caused by DA autoxidation (Krasnova and Cadet, 2009). Excessive release of DA in the synaptic cleft can cause the loss of the synaptic termini of dopaminergic neurons. DAT inhibitors have been shown to inhibit METH-induced DA release and thus have a protective effect on synaptic terminals (Shaerzadeh et al., 2018). Increased DA release in the synaptic cleft also induces apoptosis of postsynaptic neurons by activating D1 and D2 receptors, and this effect is inhibited by D1 and/or D2 receptor antagonists (Xu et al., 2005). Antioxidants help to alleviate nerve damage caused by METH and are neuroprotective (Imam et al., 2001). Protein kinase Cδ (PKCδ) is also involved in METH-induced oxidative stress and dopaminergic neurotoxicity. Inhibition of PKCδ activity can prevent METH-induced neurotoxicity (Wen et al., 2016).

Mitochondrial dysfunction is another mechanism of METH neurotoxicity. METH causes adenosine triphosphate (ATP) depletion and mitochondrial complex II inhibition. Mitochondrial complex substrates (decylubiquinone or nicotinamide) have been shown to attenuate METH-induced striatal dopaminergic neuron damage (Stephans et al., 1998; Brown et al., 2005).

The inflammatory response of the central nervous system induced by METH is a complex, interactive, and regulated process. This may be related to the symptoms of mental disorders caused by METH (Huckans et al., 2015). Microglia, astrocytes, and a series of inflammation-related factors form a network of cascade pathways. When the inflammatory response is overactivated, microglia and astrocytes can regulate each other through inflammatory cell mediators and jointly regulate changes in other factors in the inflammatory pathway. The increase in inflammatory factors can stimulate or induce microglia and astrocytes (Potula et al., 2010; Liu et al., 2012; Chen et al., 2015; Lloyd et al., 2017). Signal changes in nuclear factor-κB (NF-κB) acts as a ‘local pivotal factor’ in the control of midstream inflammation, controlling the activation of many inflammatory pathways (Mitchell and Carmody, 2018). Upstream factors, such as toll-like receptor-4 (TLR4), signal transducer and activator of transcription 3 (STAT3), extracellular signal-regulated kinase (ERK), serine-threonine kinase (AKT), and phosphatidylinositol 3-kinase (PI3K), signal changes in NF-κB signalling (Park et al., 2017), NF-κB activation, and transcription of various inflammatory cytokines, such as IL-1β, IL-6, and TNF-α, thereby mediating the cellular inflammatory response (Hou et al., 2017). D2-deficient brain regions show a significant inflammatory response. D2 agonists inhibit NF-κB phosphorylation and downstream inflammatory cytokine and chemokine production (Han et al., 2017). Due to the complex environment of the central nervous system, there are still many upstream and downstream factors of inflammation-related pathways, and their relationships have not been fully elucidated and need to be further studied.

### Candidate Chinese Herbal Medicine of Methamphetamine Abuse

METH abuse, addiction, and the resulting mental symptoms have become an increasingly important problem to be solved. Unfortunately, because the specific neurobiological mechanisms involved are complex and have not been fully and systematically elucidated, to date, there is no established pharmacotherapy for METH abuse (Siefried et al., 2020).

**FIGURE 1:**
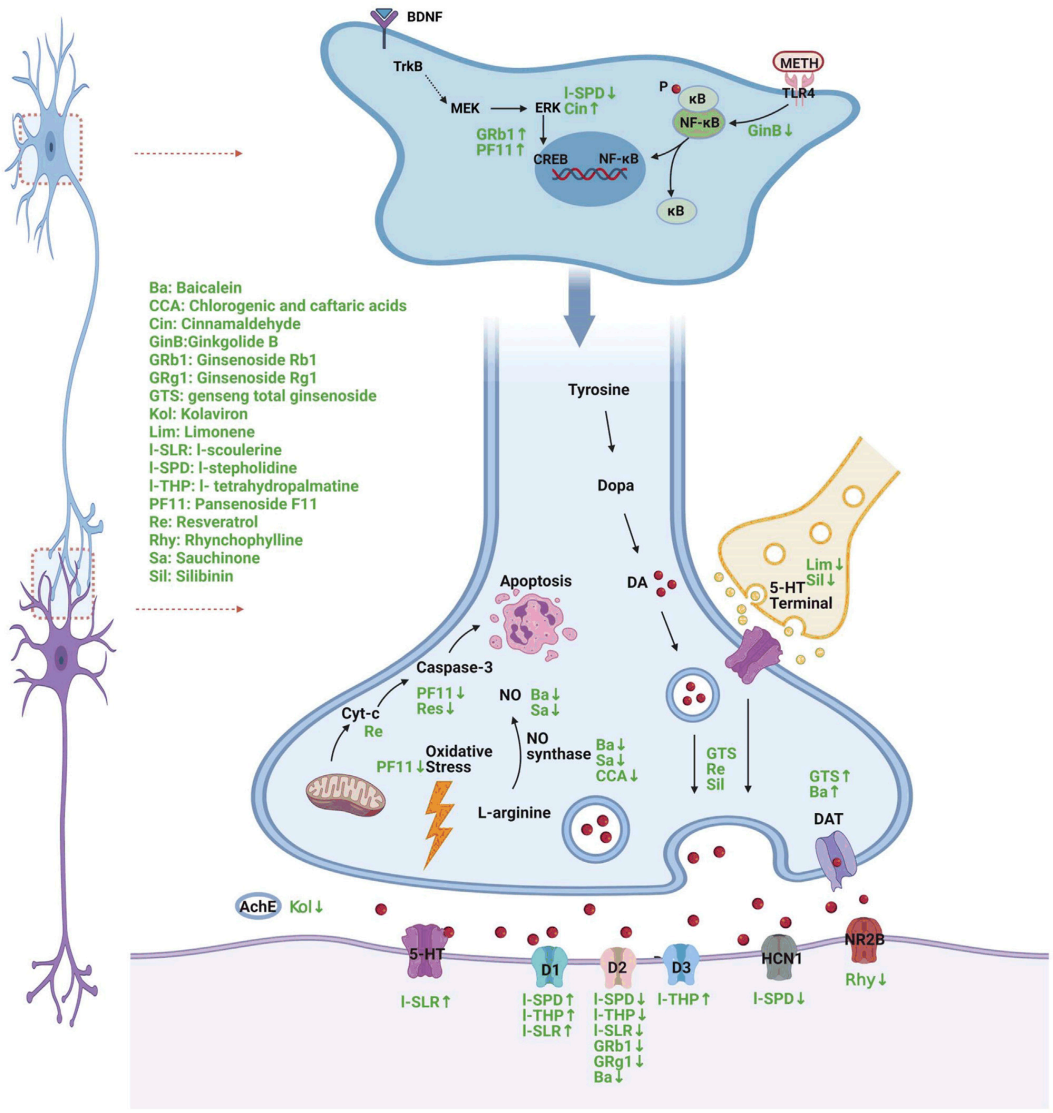
A diagram of the mechanism of different Chinese herbs in the treatment of METH-induced neuropsychiatric injury (Created with BioRender.com). ®↓ symbol indicates decrease and ↑ symbol indicates increase following METH treatment.

In recent years, research on the therapeutic effect of Chinese herbal medicine on METH has received increasing attention and has made remarkable progress. Studies have found that a variety of Chinese herbal medicines have significant therapeutic effects on psychiatric symptoms, such as addiction, depression, and cognitive impairment, induced by METH abuse and have the advantage of multitarget comprehensive treatment. We conducted a systematic review to provide new perspectives and ideas for the prevention and treatment of METH abuse. The detailed neural mechanism is illustrated in Figure 1.

### Tetrahydroprotoberberines

*Tetrahydroprotoberberines* (THPBs) are a series of alkaloids isolated from the traditional Chinese analgesic drug Yanhuasol that act on the dopaminergic system of the CNS and have a far-reaching effect (Jin and Sun, 1995). *l*-Tetrahydropalmatine (*l-*THP) and *l*-stepholidine (*l*-SPD) are members of the THPB family. They have unique pharmacological characteristics as D1 receptor agonists and D2 receptor antagonists (Jin et al., 2002) and have been proven to have value in the clinical treatment of drug dependence (Chu et al., 2008).

*l*-THP acts in the brain and induces the release of endogenous opioids such as endorphins, enkephalins, and kephalins. This may be the underlying mechanism for treating drug dependence (Jin and Sun, 1995). The reward effect in the process of METH addiction is mainly caused by the increase of DA, and the blocking effect of *l*-THP on the DA receptor in the reward system can weaken the reward effect of METH, reduce the euphoria it produces, and finally reduce the mental dependence on METH (Jin and Sun, 1995). Our other study also found that *l*-THP inhibits METH self-administration and reinstatement in rats (Gong et al., 2016). *l*-THP can suppress METH-induced rewarding in CPP mice (Su et al., 2013), and the *l*-THP inhibitory effect may be associated with the inhibition of ERK phosphorylation in the NAc and PFC (Su et al., 2020). *l*-THP inhibits METH-induced behavioral sensitization by upregulating 5-HT neuronal activity and increasing the expression of the D3 receptor (Yun, 2014a). *l*-THP treatment also has a potential protective role on METH-induced spatial memory impairment in mice (Cao et al., 2018).

*l*-SPD, a partial agonist of the D1 receptor and antagonist of the D2 receptor (Natesan et al., 2008), has a therapeutic effect on memory damage induced by METH. *l*-SPD can also attenuate METH-induced locomotor sensitization behavior in a dose-dependent manner (Ma et al., 2014). A recent study proved that *l*-SPD alleviates memory deficits in Alzheimer’s disease rats by affecting dopaminergic pathways and synaptic plasticity (Hao et al., 2015). Our previous study also demonstrated that *l*-SPD pre-treatment rescues METH-induced memory deficits by suppressing upregulated HCN1 channels and the dopaminergic pathway (Zhou et al., 2019).

*l*-scoulerine (*l*-SLR, an *l*-SPD analogue) is not only a D1 receptor agonist and D2 receptor antagonist but also a 5-HT1A receptor partial agonist. Pre-treatment with *l*-SLR reduces the chronic behavioral sensitization and METH-induced expression of CPP in mice. *l*-SLR also inhibits the anxiety-like behaviors induced by METH in zebrafish (Mi et al., 2016).

The effects of these traditional Chinese medicine (TCM) THPBs are summarized in Table 2.

**TABLE 2:** Summarized effects of TCM Corydalis and Stephania therapy on METH abuse and other psychiatric symptoms.

Herb	Compound	Types of functional impairment	Symptoms and experiment	Animal	Effective dose	Receptor or signaling pathway molecule	Author [Ref.]
*Corydalis and Stephania*	*l-*THP	METH dependence	METH self-administration and METH-induced reinstatement	Rat	*l-*THP 5 mg/kg	DA receptor	Gong et al. (2016)
			CPP	Mice	*l*-THP (10 and 20 mg/kg)		Su et al. (2013)
					*l*-THP 10 mg/kg	ERK phosphorylation	Su et al. (2020)
		Behavioral sensitization	Locomotor activity	Rat and mice	*l*-THP (10 and 15 mg/kg)	5-HT and D3 receptor	Yun (2014a)
		Memory and cognitive function impairment	Spatial memory impairment	Mice	*l*-THP (10 and 20 mg/kg)	DA receptor	Cao et al. (2018)
	*l-*SPD	Behavioral sensitization	Locomotor sensitization behavior	Rat	*l*-SPD (5 and 10 mg/kg, i.p.)	DA receptor	Ma et al. (2014)
		Memory and cognitive function impairment	Memory deficits	Mice	*l*-SPD (10 mg/kg, i.p.)	Dopaminergic pathway and HCN1 channels	Zhou et al. (2019)
	*l-*SLR	Behavioral sensitization	Behavioral sensitization	Mice	*l*-SLR (5 mg/kg)	D2 receptor antagonist, D1 receptor agonist	Mi et al. (2016)
		METH dependence	CPP				
		Anxiety-like behaviors	Anxiety-like behaviors	Zebrafish		5-HT1A receptor partial agonist	

METH = methamphetamine; 5-HT = 5-hydroxytryptamine; D or DA = dopamine; ERK = extracellular-regulated kinase; HCN1 = hyperpolarization-activated and cyclic nucleotide-gated cation 1; l-THP = l-tetrahydropalmatine; l-SPD = l-stepholidine; l-SLR = l-scoulerine; CPP = conditioned place preference.

### Ginsenoside

Ginseng is a perennial, succulent root and a family of plants known as araliaceae. Ginseng is mainly divided into American ginseng panaxquinquefolium and Korean ginseng panax ginseng. Over many years, in traditional medicine, ginseng has played a positive role in invigorating qi, calming nerves, and enhancing immunity. It has also been used as an anxiolytic, antidepressant, and memory enhancer (Lacaille-Dubois and Wagner, 1996). Ginseng prevents morphine-, cocaine-, and METH-induced tolerance and dependence in rodents (Tokuyama and Takahashi, 2001; Kim et al., 2005). Ginseng also reduces the hyperstimulation induced by METH and cocaine even after discontinuation for 30 days (Tokuyama et al., 1996).

It has been suggested that ginseng total saponin (GTS) attenuates hyperlocomotion and CPP induced by METH in rodents (Kim et al., 1996; Tokuyama and Takahashi, 2001; Kim et al., 2005). GTS modulates the activity of the dopaminergic system by reducing DA reuptake and then affecting brain DA concentrations (Lacaille-Dubois and Wagner, 1996).

Pansenoside F11 (PF11) is a special ginsenoside that is found in American ginseng but not in Korean ginseng. PF11 can reduce DA levels by regulating dopaminergic and GABA neurons in the NAc and thus exerts an inhibitory effect on METH addiction-induced behavior (Fu et al., 2016). PF11 has a neuroprotective effect and can antagonize the neurotoxic effects caused by METH addiction (Wu et al., 2003).

Ginsenoside Re can effectively prevent METH-induced mitochondrial dysfunction, oxidative damage, microglial activation, activation of proapoptotic factors, and degeneration of dopaminergic neurons by inhibiting the PKCδ gene (Shin et al., 2014; Nam et al., 2015). Both single and repeated administration of ginsenosides Rb1 and Rg1 (major components of GTS) inhibit the behavioral sensitization and CPP induced by METH (Kim et al., 1998).

One of the clinical indications of ginseng is antidepressant effects (Kennedy and Scholey, 2003). In forced swimming tasks, PF11 shortens METH-induced long periods of immobility and increases the incubation period of the Morris water maze task, suggesting that PF11 alleviates memory decline and depression-like behavior (Wu et al., 2003). A study found that ginseng saponin Rb1 has an antidepressant effect associated with the BDNF- tyrosine kinase B (TrkB) signaling pathways and that the combination of BDNF and TrkB regulates PI3K through at least three intracellular signal transduction pathways. These different signal transduction pathways ultimately regulate cell proliferation, differentiation, and apoptosis through the cyclic adenosine monophosphate response element binding protein (CREB)-dependent activation of transcription factors and are critical to play an antidepressant role (Lee et al., 2016; Kang et al., 2019). Therefore, ginseng (saponin) is also a potential candidate drug for METH-induced depression.

The effects of the TCM ginseng are summarized in Table 3.

**TABLE 3:** Summarized effects of TCM ginseng therapy on METH abuse and other psychiatric symptoms.

Herb	Compound	Types of functional impairment	Symptoms and experiment	Animal	Effective dose	Receptor or signaling pathway molecule	Author [Ref.]
*Ginseng*	GTS	Behavioral sensitization	Hyperlocomotion	Mice	200 mg/kg	Modulated reuptake of dopamine and complex pharmacological actions between dopamine receptors and a serotonergic/adenosine A2A/delta-opioid receptor	Tokuyama et al. (1996)
		METH dependence	CPP				
	Ginsenoside Rb1 and Rg1	Behavioral sensitization	Hyperlocomotion	Mice	100 and 200 mg/kg, respectively	Postsynaptic DA receptors	Kim et al. (1998)
		METH dependence	CPP		100 mg/kg, respectively		
	Ginsenoside Rb1	Anxiety-like behaviors and stress	Immobilization stress	Rat	40 mg/kg	BDNF - TrkB signaling pathways	Kang et al. (2019)
			Anxiety-like responses and post-traumatic stress		30 mg/kg	CREB	Lee et al. (2016)
	Pansenoside F11	METH dependence	CPP	Mice	4 or 8 mg/kg/day p.o	Reduce DA level by regulating dopaminergic and GABA neurons	Fu et al. (2016)
		Neurotoxic	Neurotoxic		4 and 8 mg/kg, p.o., two times at 4 h intervals, 60 min prior to METH administration	Neuroprotective	Wu et al. (2003)
		Depression	Prolonged immobility time in the forced swimming task				
		Memory and cognitive function impairment	Increased latency in morris water maze task				
	Ginsenoside Re	Neurotoxic	Oxidative damage, mitochondrial dysfunction	Mice	10 and 20 mg/kg, p.o., twice a day, 8 or 19 days	PKCδ gene	Shin et al. (2014)
			Microglial activation and dopaminergic degeneration	Human neuroblastoma dopaminergic SH-SY5Y cell lines	100 μM		Nam et al. (2015)

METH = methamphetamine; 5-HT = 5-hydroxytryptamine; D or DA = dopamine; GABA = gamma-aminobutyric acid; BDNF = brain derived neurotrophic factor; TrkB = tyrosine kinase B; CREB = cAMP-response element binding protein; PKC = protein kinase C; GTS = ginseng total saponin; CPP = conditioned place preference

### Others

*Scutellariae baicalensis Georgi* (*Huang Qin*) belongs to the labiaceae family. It has many clinical therapeutic effects. Baicalein is an active ingredient isolated from *Huang Qin* roots that has anti-inflammatory and free radical scavenging effects. Studies have shown that baicalein has a powerful neuroprotective effect (Sowndhararajan et al., 2017). A recent study confirmed that baicalein ameliorates METH-induced memory loss and amnesia through D2 receptors in mice. Baicalein also reduces METH-induced hippocampal lipid peroxidation and peroxynitrite production in mice (Wong et al., 2014). Baicalein attenuates the loss of DAT (Wu et al., 2006) and affects the DA concentration in METH-intoxicated mice in a dose-dependent manner (Liu et al., 2006). In the striatum, baicalein protects neurons from METH-induced reductions in NO content (Liu et al., 2006).

*Uncaria alkaloids* are commonly used in TCM (Shi et al., 2003). In the central nervous system, rhynchophylline has anti-convulsive, sedative, memory repair, and anti-epileptic effects (Li et al., 2015). Rhynchophylline is a noncompetitive NMDA receptor antagonist and a calcium channel blocker. It can reduce the CPP behavior of animals by reducing the expression of NR2B protein and thus reduces psycho-dependence after METH abuse (Li et al., 2014).

Hispidulin, the active constituent of the *C. inerme* ethanolic group, also decreases hyperlocomotion induced by METH (Chen et al., 2012; Huang et al., 2015). Hispidulin inhibits METH-induced behavioral sensitization, possibly by activation of the GABAA receptor α6 subunit (Liao et al., 2016).

Kolaviron, the biflavone complex in kola seeds, alleviates the stereotypical behavior induced by a single dose of METH in mice and alleviates the negative effects of METH on learning and memory. Brain histological studies also show that kolaviron preconditioning protects the hippocampus from METH-induced neurotoxicity. Kolaviron may restore METH-induced cognitive impairment by inhibiting acetylcholinesterase (Ijomone and Obi, 2013).

Possible protective effects of sauchinone against METH abuse have also been discussed. Sauchinone attenuates METH-induced dopaminergic nerve terminal degeneration. In addition, sauchinone reduces glial cell activation and inhibits the synthesis of NO through the suppression of NO synthase (Jang et al., 2012). Kim and coworkers found that sauchinone shows a dose-dependent protective effect, inhibiting the expression and acquisition of CPP induced by METH (Kim et al., 2013).

Chlorogenic acid and caftaric acid can eliminate hepatotoxicity and reverse the increase in oxidative stress induced by METH (Koriem and Soliman, 2014). Resveratrol also reduces METH-induced DA overload in the brains of rats (Miller et al., 2013). Resveratrol has a protective effect on METH-induced caspase-3-dependent apoptosis (Kanthasamy et al., 2011). Limonene reduces METH-induced hyperlocomotion in a dose-dependent manner (Yun, 2014b).

Ginkgolide B inhibits microglial cell activation induced by METH, possibly through the TLR4-NF-κB signaling pathway (Wan et al., 2017). Silibinin can reduce cognitive impairment and decrease DA and 5-HT associated with METH abuse (Lu et al., 2010). Barakol, the main component of *cassia seeds*, reduces hyperactivity induced by METH in a dose-dependent manner by inhibiting dopaminergic receptors (Sukma et al., 2002). Cinnamaldehyde can reduce METH-induced nerve damage and enhances learning and cognitive abilities through activation of the ERK pathway in the PFC (Saeed et al., 2018).

The effects of other TCM are summarized in Table 4.

**TABLE 4:** Summarized effects of other TCM therapy on METH abuse and other psychiatric symptoms.

Herb	Compound	Types of functional impairment	Symptoms and experiment	Animal	Effective dose	Receptor or signaling pathway molecule	Author [Ref.]
*Scutellariae baicalensis Georgi*	Baicalein	Memory and cognitive function impairment	Memory deficits, amnesia	Mice	1 mg/kg	D2 receptors	Wong et al. (2014)
*Uncaria alkaloids*	Rhynchophylline	Neurotoxic	Oxidative damage	Mice	1 mg/kg	Dopamine transporter	Liu et al. (2006)
		Neurotoxic	Dopaminergic neurotoxicity				
		METH dependence	Dopaminergic neurotoxicity		1 mg/kg	Elevated NO level	Liu et al. (2006)
			CPP		40 and 80 mg/kg	Reduce NR2B expression	Li et al. (2014)
*Clerodendrum inerme*	Hispidulin	Behavioral sensitization	Hyperlocomotion	Mice	10,30, and 100 mg/kg, ip; 10 nmol, intracerebellar microinjection (i.c.b.)	Activate GABAA receptors	Liao et al. (2016)
*G. kola seeds*	Kolaviron	Behavioral sensitization	Stereotypic behaviors	Rat	200, 400, and 800 mg/kg, po., 4 weeks	Inhibition of acetylcholinesterase	Ijomone and Obi (2013)
*Saururus chinensis*	Sauchinone	Memory and cognitive function impairment	Negative effects of METH on learning and memory	Mice	10 mg/kg, po	Degeneration of dopaminergic nerve terminals, NO synthase	Jang et al. (2012)
		Neurotoxic	Neurotoxicity				
		Neurotoxic	Attenuated the METH-induced degeneration of dopaminergic nerve terminals, reduced the glial cell activation, inhibited the synthesis of NO				
*Coffee beans*	Chlorogenic and caftaric acids	METH dependence	CPP	Rat	10 mg/kg, ip	NO synthase inhibitor	Kim et al. (2013)
		Oxidative stress	Oxidative stress		60 mg/kg chlorogenic acid and 40 mg/kg caftaric acid	Antioxidant stress	Koriem and Soliman (2014)
*Grape*	Resveratrol	Behavioral sensitization	Dopamine overflow	Rat	Repeated resveratrol treatment (1–20 mg/kg)	Reduce DA release	Miller et al. (2013)
*Lemon*	Limonene	Behavioral sensitization	Neuron apoptotic	Neuronal N27 cell lines	10 µM	Caspase-3 dependent pathway	Kanthasamy et al. (2011)
			Hyperlocomotion	Rat and mice	200, 400, and 600 mg/kg, i.p	5-HT neuronal function and DA release	Yun (2014b)
*Ginkgo biloba*	Ginkgolide B	Nerve inflammation	Microglial activation	BV2 cells lines	120–240 µM	TLR4-NF-κb signaling pathway	Wan et al. (2017)
*Silybum Marianum*	Silibinin	Memory and cognitive function impairment	Cognitive deficits	Mice	200 mg/kg, po.,qd, 7 days	DA and 5-HT system	Lu et al. (2010)
*Cassia siamea Lamk*	Barakol	Neurotoxic	Decreases of DA and 5-HT	Mice	100 mg/kg, ip	Dopaminergic receptors	Sukma et al. (2002)
		Behavioral sensitization	Hyperlocomotion				
*Cortex cinnamomi*	Cinnamaldehyde	Neurotoxic	Neurotoxicity	Rat	40 mg/kg, ip	ERK pathway	Saeed et al. (2018)
		Memory and cognitive function impairment	Learning and cognition deficits				

METH = methamphetamine; NO = nitric oxide; 5-HT = 5-hydroxytryptamine; D or DA = dopamine; GABA = gamma-aminobutyric acid; NR2B = N-methyl-d-aspartate receptor subtype-2B; ERK = extracellular-regulated kinase; TLR4-NF-κB = toll-like receptor 4-nuclear factor-κB; CPP = conditioned place preference

## Discussion

METH dependence and its related neurological and psychiatric problems involve multiple transmitter systems in multiple brain regions. METH dependence is caused by very complex mechanisms involving DA, Glu, 5-HT, acetylcholine, and GABA. With a history of more than 200 years, TCM drug rehabilitation has accumulated rich experience and formed a set of unique theories and methods. The treatment philosophy of TCM drug abstinence is to support healthy qi and eliminate toxic drugs, and this philosophy highlights the characteristics of treatment based on syndrome differentiation. More importantly, TCM compounds also have multitarget effects that are similar to cocktail therapy, aiming to address METH substance dependence and the neuropsychiatric problems derived from this complex multitarget intractable encephalopathy. TCM can be regarded as a useful attempt and exploration. Unfortunately, only a few TCM or chemical constituents have sufficient literature to identify promising candidates for METH abuse. Further basic and clinical studies are needed.
